# Optimizing surface properties and particle morphology for metal ion adsorption: precise tuning *via* Pickering emulsion polymerization†

**DOI:** 10.1039/d5na00417a

**Published:** 2025-07-03

**Authors:** Andrei Honciuc, Oana-Iuliana Negru, Mirela Honciuc

**Affiliations:** a “Petru Poni” Institute of Macromolecular Chemistry 41A Gr. Ghica Voda Alley Iasi 700487 Romania honciuc.andrei@icmpp.ro

## Abstract

Tuning the chemistry and morphology of polymer microparticle adsorbents for metal ion adsorption is a complex synthetic challenge. Recently, Pickering Emulsion Polymerization Technology (PEmPTech) has emerged as a straightforward and effective method, presenting a compelling alternative to traditional synthesis techniques. In this study, we explore the versatility of PEmPTech by fine tuning of the composition and morphology of the polymer microspheres to maximize their adsorption capacity for metal ions. By systematically varying the amounts of crosslinker, monomer and porogen, we generated several homologous series of microsphere adsorbents and evaluated their Cu(ii) ion removal efficiency from aqueous solutions. The data reveal a clear structure–activity relationship, highlighting the influence of crosslinker and porogen levels on the structure and morphology of the polymer microspheres and consequently on their metal ion adsorption capacity. The adsorption performance was found to decrease with increasing degree of cross-linking and to improve with higher porosity induced by the porogen. Influence of other factors on the adsorption capacity of the microparticles, such as the nature of the crosslinker, size of the microparticles, water contact angle is also discussed. This study advances our understanding of the design parameters critical for developing effective polymer microsphere adsorbents *via* PEmPTech as a sustainable method for producing high-performance adsorbents in a completely aqueous and surfactant-free environment.

## Abstract 2

Polymer microsphere adsorbents were synthesized *via* Pickering emulsion polymerization with tunable morphology and composition. Their Cu(ii) ion uptake reveals structure–activity relationships driven by crosslinking and porogen-induced porosity.

## Graphical abstract

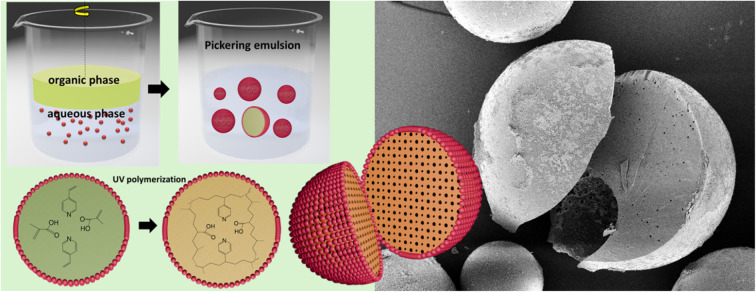

## 1. Introduction

The removal of heavy metal ion pollutants from water, whether for industrial use or consumption, is a critical environmental challenge that can be addressed through the use of adsorbent materials. A wide range of materials are available for such applications, spanning from natural, renewable material to synthetic polymers.^1^ Notably, in contrast to the bio-based adsorbents, polymer-based adsorbents offer consistent performance, high reproducibility, and the potential for rational design and optimization, thereby enhancing their efficacy in pollutant removal. However, the development of greener and more energy efficient routes for their synthesis remains an important goal. The synthesis of polymer microsphere adsorbents typically involves methods, such as suspension polymerization, precipitation from organic solvents or their mixtures,^2–4^ emulsion polymerization, swelling, seeded polymerization, or a combination thereof.^5–7^ Various strategies for preparation of polymer adsorbents have been extensively reviewed.^8–10^ Recently, we introduced^11^ the Pickering Emulsion Polymerization Technology (PEmPTech), as a green and environmentally friendly method for preparation of the polymer microsphere adsorbents for capturing metal ions.

The key advantage of Pickering emulsions lies in their stabilization by nanoparticles rather than surfactants, resulting in significantly improved emulsion stability compared to classical surfactant-stabilized systems. Moreover, PEmPTech represents a greener alternative to the more traditional precipitation and emulsion polymerization methods, as it operates in an aqueous medium without the need for organic solvents or surfactants, employing environmentally neutral silica nanoparticles as stabilizers.^12^ In Pickering emulsions, oil droplets are stabilized by the spontaneous adsorption of nanoparticles at the oil/water interface, forming a monolayer that acts as a robust barrier against coalescence. This stabilization mechanism allows the emulsions to maintain integrity even under high-temperature polymerization conditions. In this work, we demonstrate the versatility of the method with respect to tuning the composition of the microsphere adsorbents, to achieve maximum performance in terms of metal ion adsorption capacity.

In the present work, we demonstrate the versatility of PEmPTech in tuning the chemical composition and morphology of polymer microspheres to optimize their metal ion adsorption capacity. By systematically varying the amount of crosslinker and porogen, we generated homologous series of microsphere adsorbents and evaluated their efficiency in Cu(ii) ion removal from aqueous solutions. Our results reveal clear structure–activity relationships linking the levels of crosslinker and porogen to adsorption capacity. These findings provide valuable insights into the design parameters necessary for the development of effective polymer microsphere adsorbents. Furthermore, PEmPTech stands out as a sustainable, surfactant-free, and fully aqueous approach to the scalable production of high-performance adsorption materials.

## 2. Materials and methods

### 2.1. Materials

4-Vinylpyridine 95% (4-VP), incorporating the inhibitor hydroquinone (100 ppm), methacrylic acid 99% (MA), containing 4-methoxyphenol (250 ppm), ethylene glycol dimethacrylate (EGDMA) 97.5% stabilized with hydroquinone monomethyl ether, divinylbenzene (DVB), technical grade 80%, stabilized with the inhibitor monomethyl ether hydroquinone, aluminium oxide (Al_2_O_3_), tetraethylorthosilicate 99% (TEOS), and (3-glycidoxypropyl)trimethoxysilane 98% (Gly), were bought from Sigma-Aldrich (Merck, KGaA, Darmstadt, Germany). All products containing inhibitors were first purified through an Al_2_O_3_ column prior to their usage. Benzoin methyl ether (BME) 97% was purchased from ABCR; GmbH Karlsruhe, Germany. Copper chloride (II) dihydrate pure p.a. (CuCl_2_ × 2H_2_O), nickel(ii) chloride hexahydrate pure p.a. (NiCl_2_·6H_2_O) and cobalt(ii) chloride hexahydrate pure p.a. (CoCl_2_·6H_2_O) were purchased from ChemPUR Feinchemikalien und Forschung GmbH (Karlsruhe, Germany), hydrochloric acid (HCl), ≥37%, was purchased from Fluka (Honeywell Specialty Chemicals, Seelze, Germany). Toluene, and ethanol absolute 99.3% (EtOH), were bought from Chemical Company (Romania). Ammonium hydroxide (NH_4_OH) solution (28–30%) was purchased from Sigma-Aldrich (Merck KGaA, Darmstadt, Germany) EMSURE® ACS, Reag. Ph Eur.

### 2.2. Methods

#### 2.2.1. Microsphere preparation *via* Pickering emulsion polymerization

First, the inhibitors presents in the monomers were removed by passing them through basic alumina columns. For the oil-in-water (o/w) Pickering emulsion polymerization we have used surface functionalized silica nanoparticles that were synthesized according to the procedures we have previously reported.^13^

The polymer microspheres were obtained by PEmPTech method *via* polymerization of o/w Pickering emulsions. In the o/w emulsion, the oil phase is represented by the 4-vinyl pyridine (4-VP) and methacrylic acid (MA) (in volumetric ratio), crosslinking agent (DVB or EGDMA), the porogenic agent (toluene) and the UV initiator BME. The aqueous phase contains 5 mg of silica nanoparticles, for stabilizing the emulsion. The mixture was sonicated at 2000 rpm, for 3 min, using a vortex mixture LLG (lab Logistic Group GmbH, Meckenheim, Germany). Next Pickering emulsion was exposed for 2 h to irradiation by UV lamp (wavelength = 365 nm, with 4 lamps, each with an intensity = 2.2 mW cm^−2^). After de polymerization, the products were sonicated in an ultrasonic bath for 1 min, then the polymerization product was filtered and washed with 40 mL EtOH to remove the unreacted monomer and left to dry at room temperature.

#### 2.2.2. Measurement of ion extraction and recovery capacity of polymer absorbents

The metal ion concentration in the diluted supernatant and filtrate were analyzed using an UV-vis spectrophotometer (DLAB Scientific Co., Ltd., China). First, calibration curves were generated corresponding to maximum absorption wavelength *λ*_max_ = 812 nm for CuCl_2_ × 2H_2_O.

For the ion extraction, which refers to the extraction of metal ion from a stock solution, 0.5 g of polymer absorbent was dispersed in 10 mL stock solution with a known concentration, typically 3 × 10^−2^ M, when not otherwise specified.

The metal ion extraction capacity *q*_e_ (mg g^−1^) was calculated with the formula:1
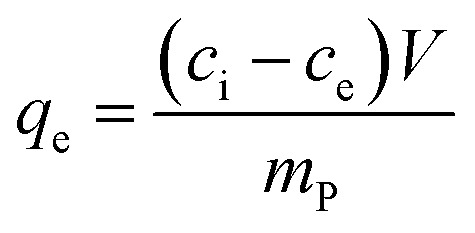
where *c*_i_ (mg L^−1^) is the initial concentration of a stock solution or the contact solution, *c*_e_ (mg L^−1^) is the extracted concentration, *V* (L) is the volume of the sample, and *m*_P_ (g) is the dry mass of the sorbent.

For the ion recovery, which refers to the recovery of metal ion from the polymer absorbent, 0.5 g of polymer absorbent was dispersed in 10 mL 5% HCl the samples were then left in this condition for approx. 12 h. The particles were separated from the aqueous medium as described previously. Supernatant and filtrate were analyzed using UV-vis, and the metal ion recovery capacity *q*_r_ (mg g^−1^) was calculated by:2
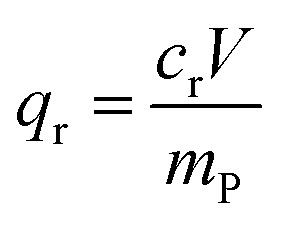
where *c*_r_ (mg L^−1^) is the concentration of metal ions recovered from the particles, *V* (L) is the volume of the sample, and *m*_P_ (mg) is the dry mass of the particles.

The procedure of extraction-recovery was repeated three or four times.

### 2.3. Polymer microsphere characterization

The 2D films were investigated with a Verios G4 UC (Thermo Fischer Scientific Inc., Eindhoven, The Netherlands) scanning electron microscope (SEM), with a 5 keV beam energy, using an Everhart-Thornley detector, beam spot 50 pA.

The porosity of the microspheres was determined from the SEM images of the cross-section of the microspheres with ImageJ™ software, by utilizing the Ferret diameter analysis protocol. The results are presented in the Fig. S1 and S2 in the ESI.†

### 2.4. Measurement of the water contact angle *via* the Washburn method

Water contact angle of the microspheres obtained by Pickering emulsion was determined *via* the capillary rise method, using the DCAT 15 Tensiometer balance (Data Physics Instruments GmbH, Filderstadt, Germany), equipped with the DCATS 32 software module for calculating the contact angle *via* Washburn method. The capillary constant of the samples was determination with hexane. Glass tubes with inner diameter of 9 mm, outer diameter of 11 mm, and length of 60 mm were used as sample holder. Approximately 0.6 g of microspheres were placed into the sample holder with manual tapping to obtained uniform packing of the microspheres. The height of the probes in each holder was approximately 20 mm. After packing, the holder was placed onto the electronic balance of the tensiometer. The weight gain of the sample holder after contact with liquids was recorded.

## 3. Results and discussions

**Fig. 1:**
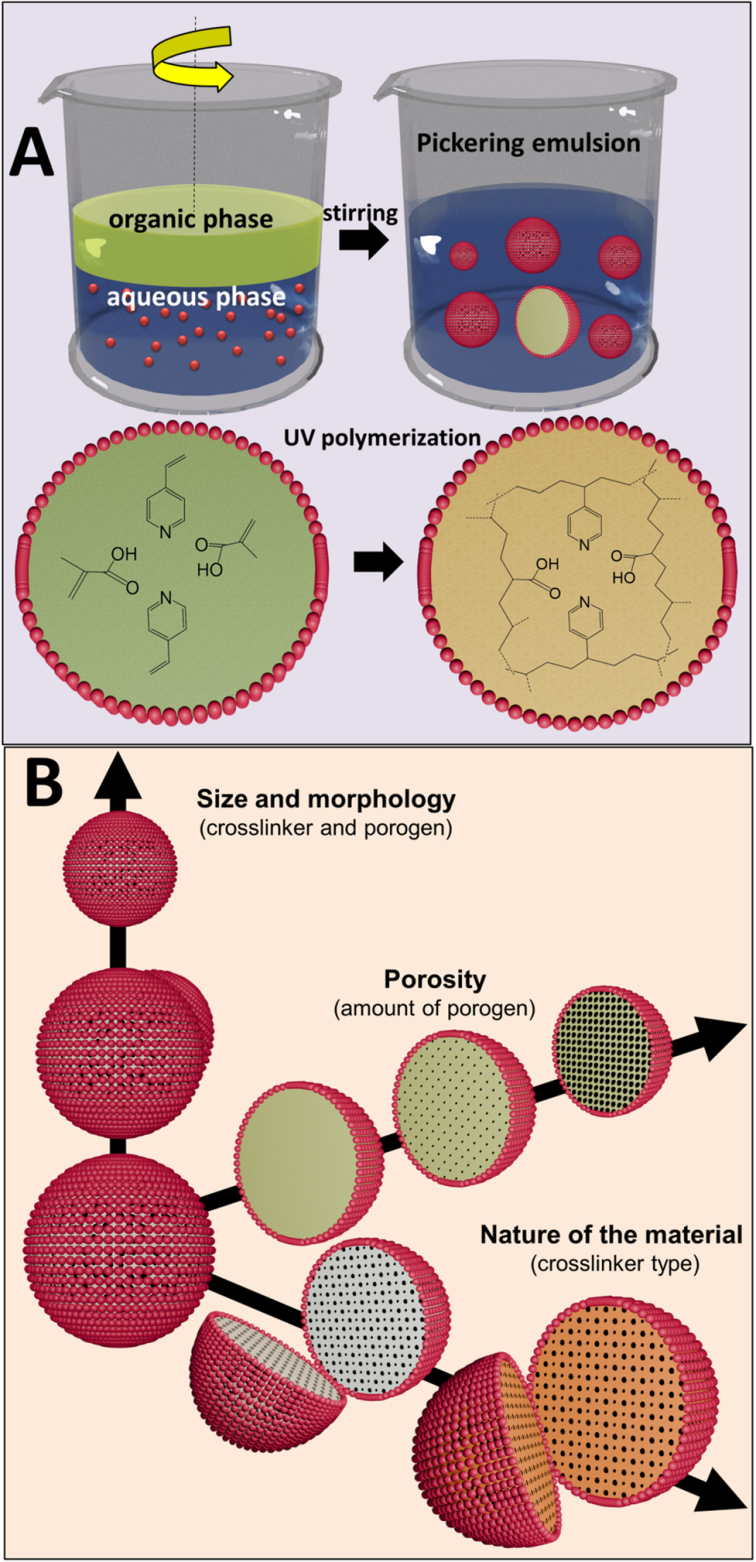
(A) Scheme depicting the formation of o/w Pickering emulsion and the conversion of monomer colloidosomes stabilized by silica nanoparticles into polymer microspheres with nanostructured surface. (B) Schematics summarizing the effect of the crosslinker and porogen towards the morphology, porosity and the nature of the polymer microspheres obtained *via* PEmPTEch.

In this work we study the effect of material composition, size and morphology, of series of polymer microspheres on the adsorption capacity toward metallic ions from water, *i.e.* Cu(ii) obtained *via* PEmPTech. The aim of this study is to uncover the role of the main parameters governing the intake capacity for ions of these adsorbents. The schematics depicting the principle of the PEmPTech method is given in Fig. 1. The process of obtaining surface nanostructured polymer microspheres begins with generating o/w Pickering emulsion, whereas the dispersed phase represent the oil phase, typically vinyl bearing monomers, a porogen solvent, and oil soluble polymerization initiator, see Fig. 1A. The Pickering emulsion droplets are also called colloidosomes because these are complex self-assembled microstructures composed of water-immiscible vinyl bearing monomers that have a monolayer of self-assembled nanoparticles adsorbed onto their surface.^14^ It has been shown previously that the Pickering emulsions are extremely stable due to irreversible adsorption of the nanoparticles at the surface of the colloidosomes and the rigidity of the monolayer formed that provide a steric-like barrier against coalescence of emulsion droplets.^15^ This makes the Pickering emulsions very stable and suitable for polymerizations given that the oil phase is polymerizable monomer. Upon polymerization all the colloidosomes are converted into polymer microspheres which have now the entire surface self-assembled nanoparticle trapped into a monolayer conferring the formed structures a nanostructured surface. The schematics presented in Fig. 1A depict the chemical structure and the morphology of the obtained nanostructured microspheres. In this work, the polymerizable oil droplets or colloidosomes are composed of vinyl pyridine and methacrylic acid, an inert monomer which plays the role of crosslinker, DVB or EGDMA, and a porogen solvent, toluene. For the preparation of the Pickering emulsions, we employed functionalized silica nanoparticles bearing glycidyl functional groups, which possess high surface energy due to their significant polar component, as reported in our previous works.^13,16^ This high surface polarity facilitates the stabilization of o/w emulsions even when using relatively polar monomers such as vinyl pyridine and methacrylic acid.

Previously, we have demonstrated that microspheres of this composition are capable of adsorbing metal ions from water, particularly Cu(ii) ions.^11^ However, no systematic studies were conducted to establish structure–activity relationships linking the morphology and size of the microparticles to their adsorption performance. In our earlier reports,^11,17^ we also provided detailed chemical characterization using FTIR spectroscopy and semi-quantitatively assessed the adsorption of Cu(ii) ions within the microparticles through SEM-EDX analysis.

It is well established that the amount and nature of the crosslinker affect particle size and morphology, while the porogen content strongly influences porosity. However, conventional synthesis methods for polymer microsphere adsorbents offer limited compositional flexibility, making structure–activity studies challenging. In contrast, PEmPTech enables broad and precise tuning of the oil phase composition. This is summarized in the scheme depicted in Fig. 1B, conceptually depicting the main parameters that will be monitored in this study, such as size, morphology, porosity and the material nature of the adsorbent microspheres by gradually changing the amount and type of crosslinker and the amount of porogen. In addition and most importantly, we investigate how these changes in morphology, porosity and nature of the material affect the capacity of the microspheres towards Cu(ii) ion adsorption.

To ensure that the measured adsorption capacities of the microparticles accurately reflect only the influence of particle morphology, the experimental conditions in this study were carefully selected. Specifically, we employed an extended adsorption time (24 hours) and used a relatively high ion concentration in the stock solution (3 × 10^−2^ M Cu(ii)), which, according to our previous reports,^11,17^ guarantees that maximum adsorption capacity is reached. Consequently, variations observed in adsorption capacities among the different microparticles directly correspond to differences in their morphology and internal structure rather than adsorption kinetics or ion concentration.

### 3.1. Synthesis of homologous series of polymer microspheres

For an in-depth study the morphology of the microspheres obtained *via* PEmPTech with the variation of several composition parameters, such as amount of crosslinker, type of crosslinker, amount of porogen, we have synthesized six homologous series of microsphere adsorbents, divided into two main groups, A and B, see Table 1. In group A the type of crosslinker we chose is the DVB, while in group B we have chosen EGDMA as a crosslinker.

Composition of the homologous series of polymer microspheres obtained with varying the amount of crosslinker DVB (series A1) and EGDMA (series B1)

**Table:** 

Group A					
Series A1	A1-6.2%	A1-11.7%	A1-16.5%	A1-20.9%	A1-24.8%
DVB (mL)	0.25	0.50	0.75	1.00	1.25
VP (mL)	1.25	1.25	1.25	1.25	1.25
MA (mL)	1.25	1.25	1.25	1.25	1.25
Toluene (mL)	0.75	0.75	0.75	0.75	0.75
DVB molar% series A1	6.2	11.7	16.5	20.9	24.8

**Table:** 

Group B					
Series B1	B1-6.2%	B1-11.7%	B1-16.5%	B1-20.9%	B1-24.8%
EGDMA (mL)	0.33	0.66	0.99	1.31	1.64
VP (mL)	1.25	1.25	1.25	1.25	1.25
MA (mL)	1.25	1.25	1.25	1.25	1.25
Toluene (mL)	0.75	0.75	0.75	0.75	0.75
EGDMA molar% series B1	6.2	11.7	16.5	20.9	24.8

#### 3.1.1. Homologous series of polymer microspheres with type and amount of crosslinker

**Fig. 2:**
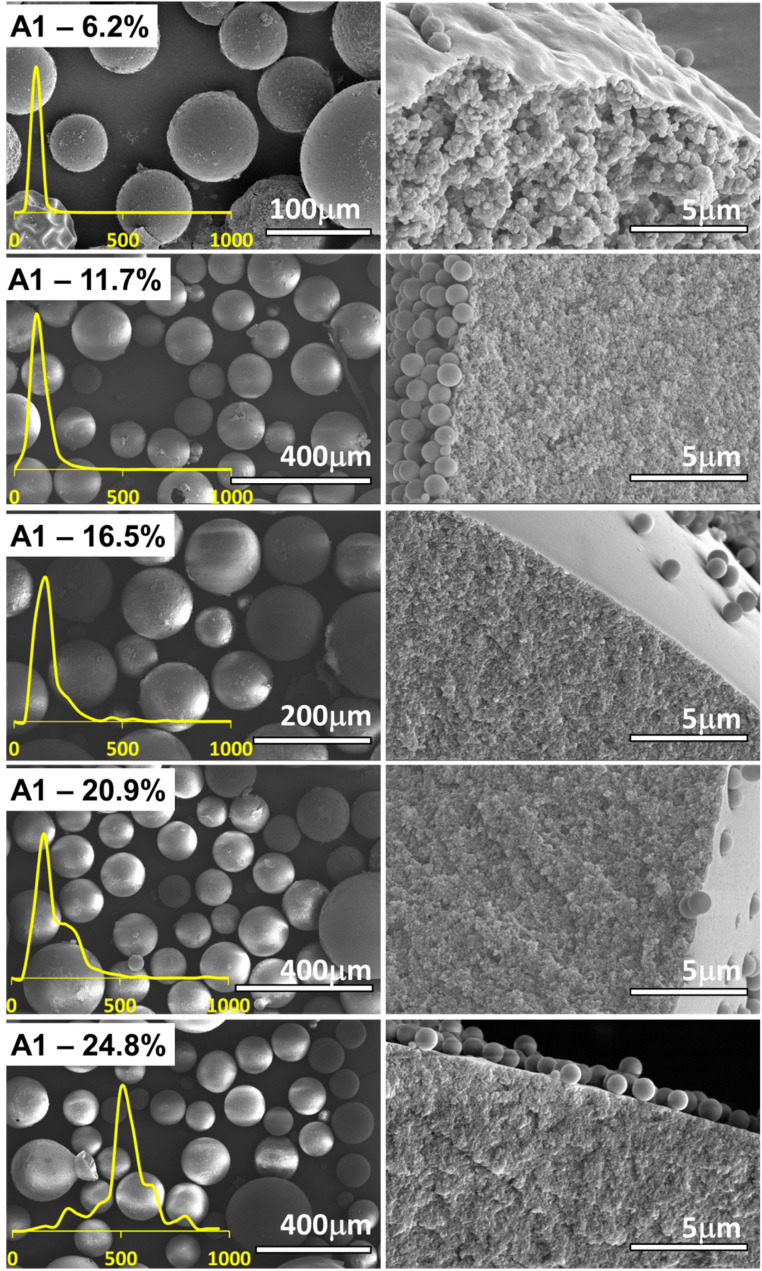
SEM images showing difference in the morphology of the polymer microspheres in the homologous series A obtained *via* PEmPTech, where the amount of crosslinker varies, from 6.2% to 24.8% DVB molar fraction. The left column of images depicts the microspheres and the right column the cross-sectional view of the microspheres. Insets show the particle size distribution for each type of microparticle.

**Fig. 3:**
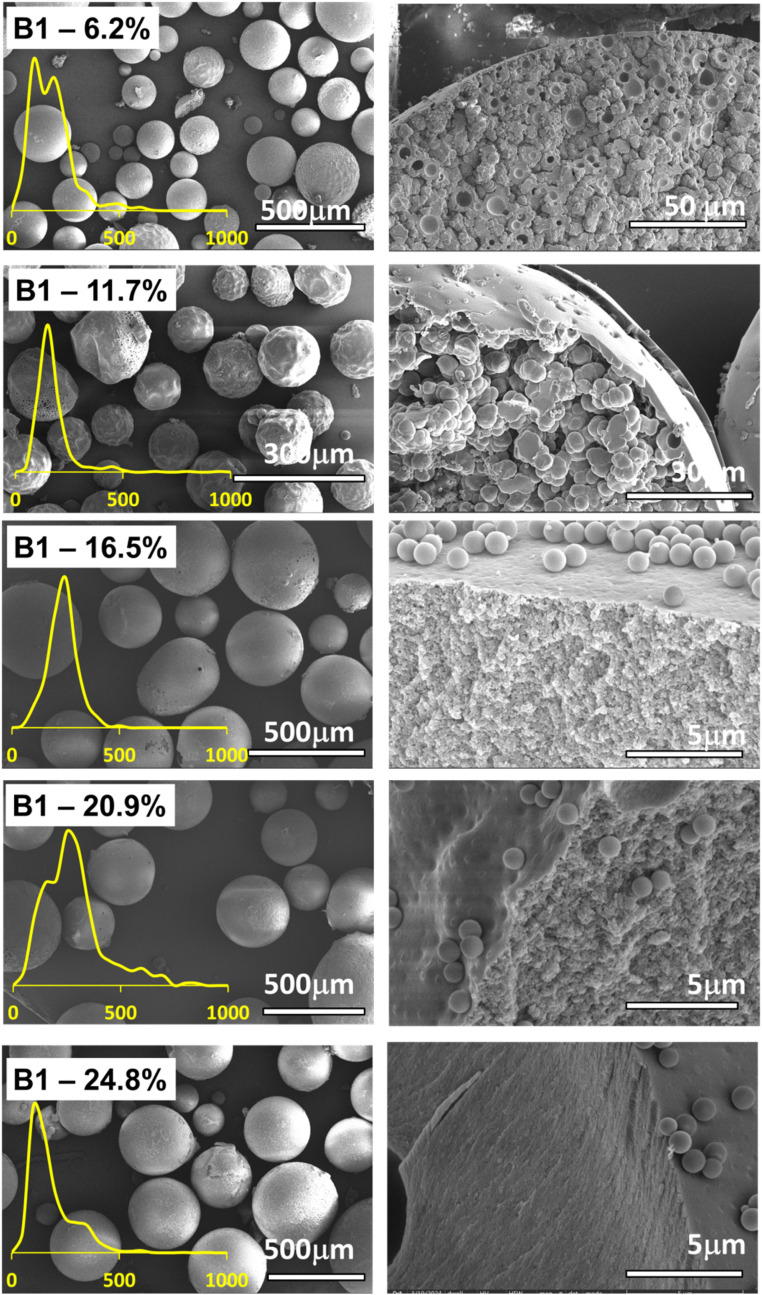
SEM images showing difference in the morphology of the polymer microspheres in the homologous series B obtained *via* PEmPTech where the amount of crosslinker varies, from 6.2% to 24.8% EGDMA. The left column of images depicts the polymer microspheres and the right column the cross-sectional view and the inner morphology of the microspheres. Insets show the particle size distribution for each type of microparticle.

To observe the effect and the amount of the crosslinker on the morphology and the characteristics of the polymer microspheres obtained *via* PEmPTech, we have chosen to vary the amount of the crosslinkers, expressed in molar percentage, while all other composition parameters, such as the monomers and porogen are kept the same throughout the synthesis, see Table 1. We chose that the amount of crosslinking monomers in the series belonging to the group A and group B varied with the same molar percentage. The Pickering emulsions were generated in the same conditions and the polymerization conditions were the same. The SEM images of the obtained microspheres for the series A1 and series B1 are presented in the in Fig. 2 and 3 respectively.

**Fig. 4:**
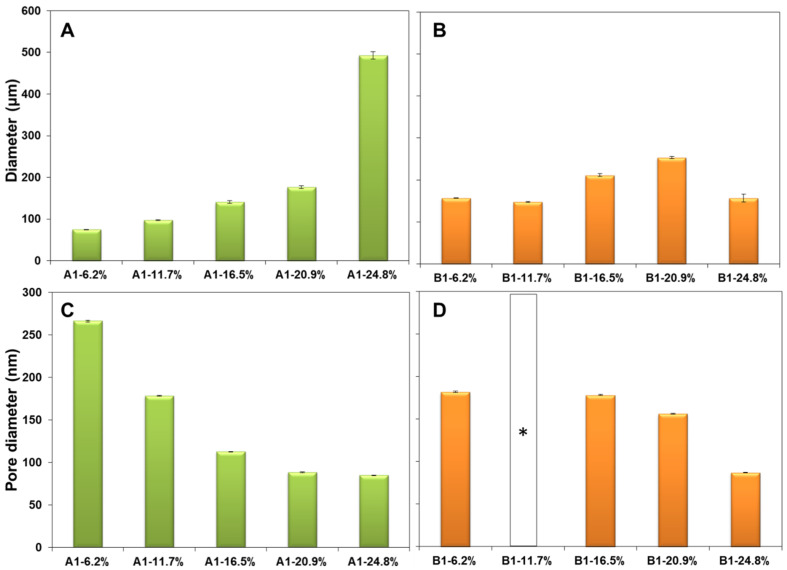
Histograms depicting the average diameter of the polymer microspheres from the (A) series A1 and (B) series B1. Histograms depicting the diameter of the pores of the (C) series A1 and (D) series B1. *For the blank bar the pore sizes could not be determined.

There are several key differences that can be observed with the nature of the crosslinker. The first observation is that with the increase in the molar percentage of the crosslinker in the composition the polymer microspheres become larger, see Fig. 4, which is expected considering that the volume of the organic phase in the Pickering emulsion to the amount of nanoparticle increases. Secondly, for the same molar composition the diameter of the polymer microspheres from series B1 are largely comparable to those from the series A1, except for the last member of the series, for 24.8% crosslinker, see Fig. 4. Clearly, at 24.8% molar fraction of EGDMA the microspheres has reached the upper limit of size due to the larger solubility in water of the EGDMA than that of the DVB, which essentially drives out the former crosslinker from the oil droplets of the emulsion.

The pore analysis of the samples from each series, Fig. 4C, D and S1 in the ESI,† reveals an inverse relationship between the pore size and the crosslinker amount, namely the increase in the amount of crosslinker decreases the pore size. This finding is rather expected and it is known that the effect of the crosslinker is to reduce the porosity of the polymer. Remarkable is the fact that the change in the pore size is much more dramatic for the series A1 than for the series B1, which is due to the fact that the DVB is a stronger crosslinker than the EGDMA. A second observation is that in both series A1 and series B1, the diameter of the obtained microspheres is inversely correlated with the pore sizes, namely with the increase in the amount of the crosslinker added, the diameter of the pores decreases, Fig. 4C and D, and the diameter of microspheres increases, Fig. 4A and B. The impact of this aspect on the capacity toward metal ion adsorption will be analyzed in the next section.

It should be noted that the porosity investigated in this work refers primarily to macroporosity, with pore sizes significantly larger than 50 nm, which are beyond the reliable detection range of nitrogen adsorption techniques such as BET analysis. Instead, the macroporosity was assessed indirectly based on the composition of the polymer formulation and the morphological features observed by SEM imaging, which reflect the internal structuring induced by variations in porogen and crosslinker content.

#### 3.1.2. Homologous series with the variation in the level of porogen

Similarly, we produced two homologous series, series A2 with constant amount of crosslinker DVB and series B2 with constant amount of crosslinker EGDMA but this time we have changed gradually the amount of toluene, according to the recipes given in Table 2. Because the toluene does not participate in the polymerization reaction, the molar fraction of the monomer does not change. Therefore, the total amount of crosslinker and ligand are considered constant throughout the homologous series A2 and series B2, while toluene increases in volume.

Composition of the homologous series of polymer microspheres obtained with constant amount of crosslinker DVB (series A2) and EGDMA (series B2) and varying the volume of toluene porogen

**Table:** 

Group A					
Series A2	A2-1	A2-0.75	A2-0.5	A2-0.25	A2-0
DVB (mL)	0.75	0.75	0.75	0.75	0.75
VP (mL)	1.25	1.25	1.25	1.25	1.25
MA (mL)	1.25	1.25	1.25	1.25	1.25
Toluene (mL)	1.00	0.75	0.50	0.25	0.00
DVB molar% series A2	16.52	16.52	16.52	16.52	16.52

**Table:** 

Group B					
Series B2	B2-1	B2-0.75	B2-0.5	B2-0.25	B2-0
EGDMA (mL)	0.99	0.99	0.99	0.99	0.99
VP (mL)	1.25	1.25	1.25	1.25	1.25
MA (mL)	1.25	1.25	1.25	1.25	1.25
Toluene (mL)	1.00	0.75	0.50	0.25	0.00
EGDMA molar% series B2	16.52	16.52	16.52	16.52	16.52

**Fig. 5:**
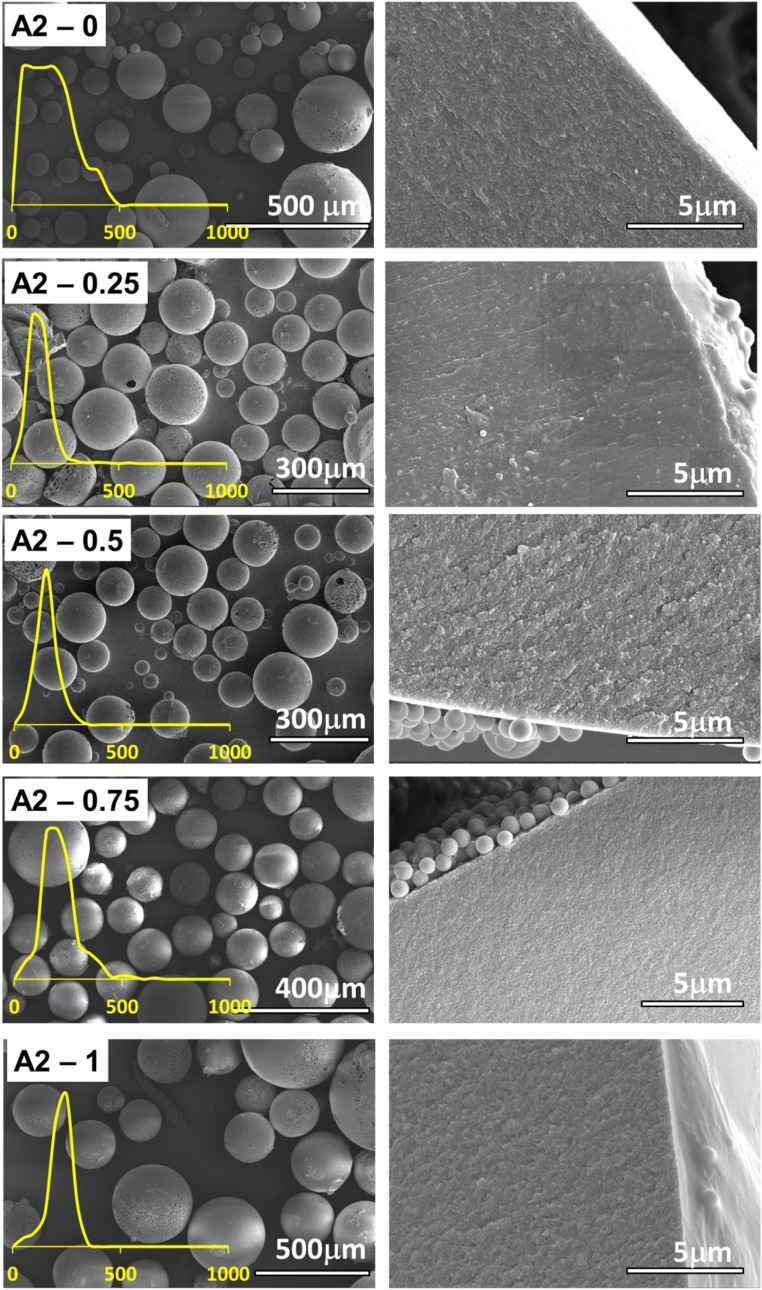
SEM image of the series A2, DVB crosslinker, with increasing amount of porogen from 0 to 1 mL toluene. The left column of images depicts the polymer microspheres and the right column the cross-sectional view and the inner morphology of the microspheres. Insets show the particle size distribution for each type of microparticle.

**Fig. 6:**
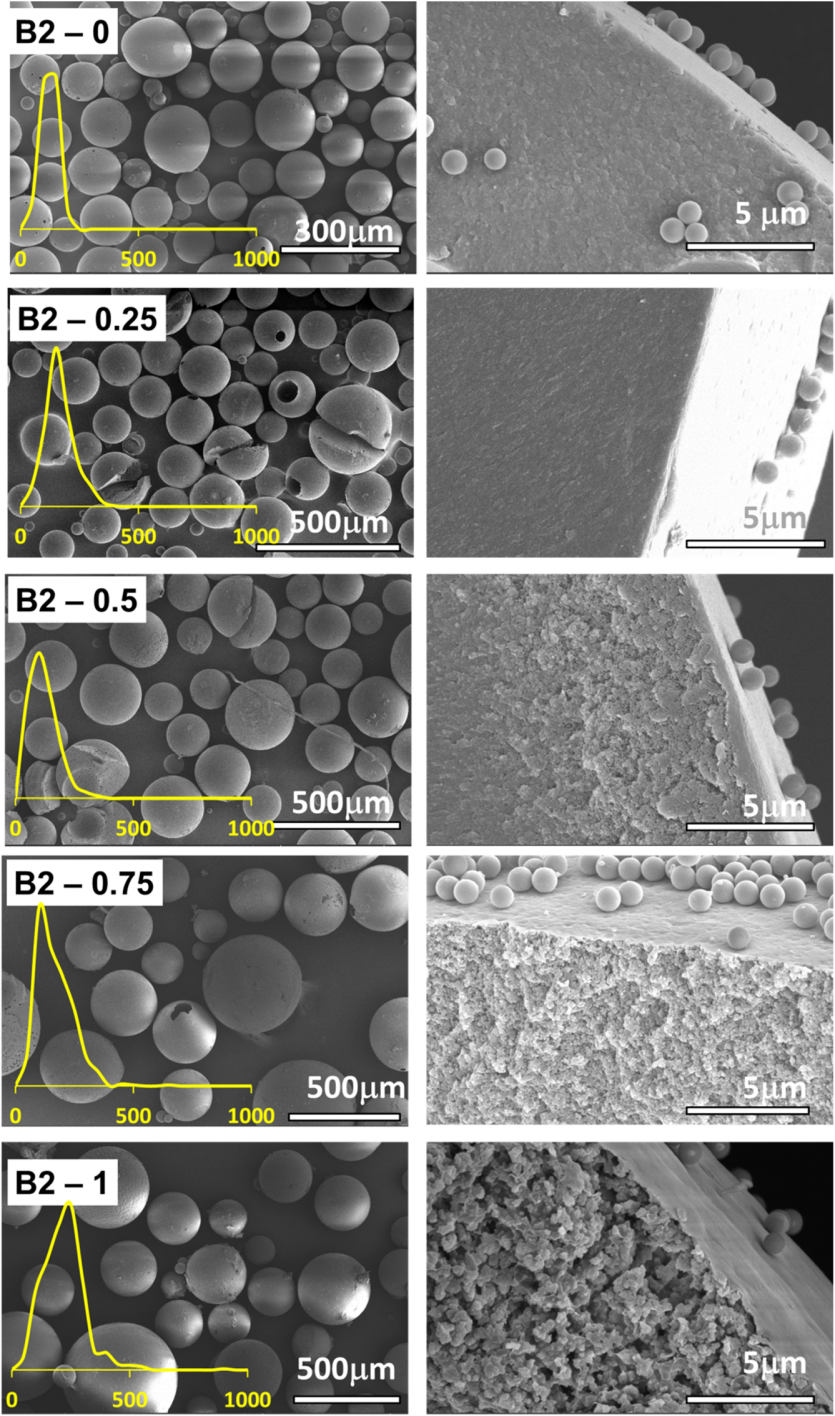
SEM image of the series B2, EGDMA crosslinker, with increasing amount of porogen from 0 to 1 mL toluene. The left column of images depicts the polymer microspheres and the right column the cross-sectional view and the inner morphology of the microspheres. Insets show the particle size distribution for each type of microparticle.

The SEM images of the series A2 are given in Fig. 5 and the SEM images of the series B2 are given in Fig. 6. The SEM images for the first and last member of each series indicate a clear morphology change. For series A2, between the microspheres with the lowest amount of porogen material show some change in the morphology in the cross-section of the microsphere, indicating a more porous structure, see Fig. 5. As to be expected toluene fulfills its role of porogen. However, even more dramatic changes can be observed in the series B2, between the microspheres with no toluene and microspheres with maximum amount of toluene, 1 mL, see Fig. 6. The microspheres with the highest amount of toluene exhibit a very porous structure, the effects observed in the series B2 are even more dramatic than in the case of series A2, signaling that indeed toluene has even more profound effects onto the morphology of the material in the former case.

**Fig. 7:**
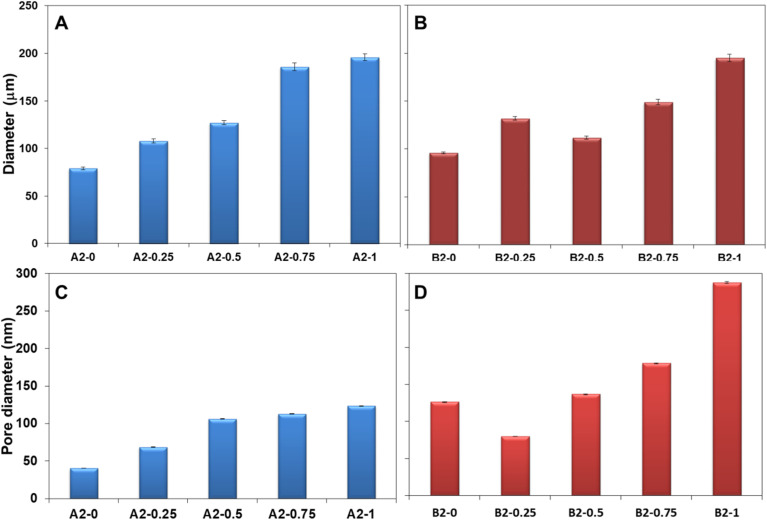
Histogram showing the evolution of the average diameters of the (A) microspheres in the series A2 and (B) microspheres in the series B2. Histogram showing the evolution of the average diameter of the nanopores in the microspheres for the series A2 (C) and series B2 (D). The error bars represent the standard error from at least 500 microspheres.

Increase in the amount of toluene immediately impacts the average diameter of the polymer microspheres from the Pickering emulsion polymerization. For example, in the series A2, the average diameter increases in the series from the 70 μm to about 180 μm, so more than double, see Fig. 7A. For series B2 the same effect can be observed Fig. 7B whereas the average diameter almost doubles in the homologous series.

The pore analysis for the samples in the series A2 and series B2 reveal that indeed the pore diameter increases with the increase in the amount of toluene porogen utilized, see Fig. 7C, D and S2 in the ESI.† It can be noted that on average, the pore sizes evolves such that it follows a steeper slope in the series B2, with pores diameter increasing from 125 nm to 280 nm, than in the series A2, with pore diameters increasing from 40 nm to 120 nm. In addition, the pore size evolution in the series A2 and B2, Fig. 7C and D, correlates directly with the diameters of the microspheres, Fig. 7C and D. The impact of these structural changes towards the capacity for metal ion adsorption will be analyzed in the next section.

### 3.2. Effect of composition on the absorption capacity of the polymer microspheres for Cu(ii) ions

To accurately assess how variations in the microparticle composition affect their adsorption capacity toward Cu(ii) ions, the experimental conditions were specifically chosen to eliminate kinetic and concentration-related dependence. Thus, an extended adsorption period (24 hours) and a relatively high initial Cu(ii) concentration (3 × 10^−2^ M) were utilized, conditions previously demonstrated^11,17^ to guarantee that maximum adsorption capacities are achieved. Additionally, as shown in Tables 1 and 2, the molar fraction of the ligand remains constant across all microparticle compositions, ensuring that variations in the adsorption performance observed in this study directly reflect differences in particle morphology and internal structure, rather than changes in ligand–ion interactions. While the amount of ligand that is the sole component capable of binding metal ions remains constant for all the recipes in each of the series synthesized, this work aims at highlighting the influence of the non-metal ion binding components and the role they play. To the best of our knowledge this work is the first to observe such a precise adjustment of the adsorption capacity for adsorbent polymer microspheres created *via* Pickering emulsion polymerization method. Thus, we begin our exploration with the adsorption capacities for the series A1 and series B1 for which the level of porogen is constant at 0.75 mL, but the amount of crosslinker increases monotonously in the series from 6.2% to 28.4%.

**Fig. 8:**
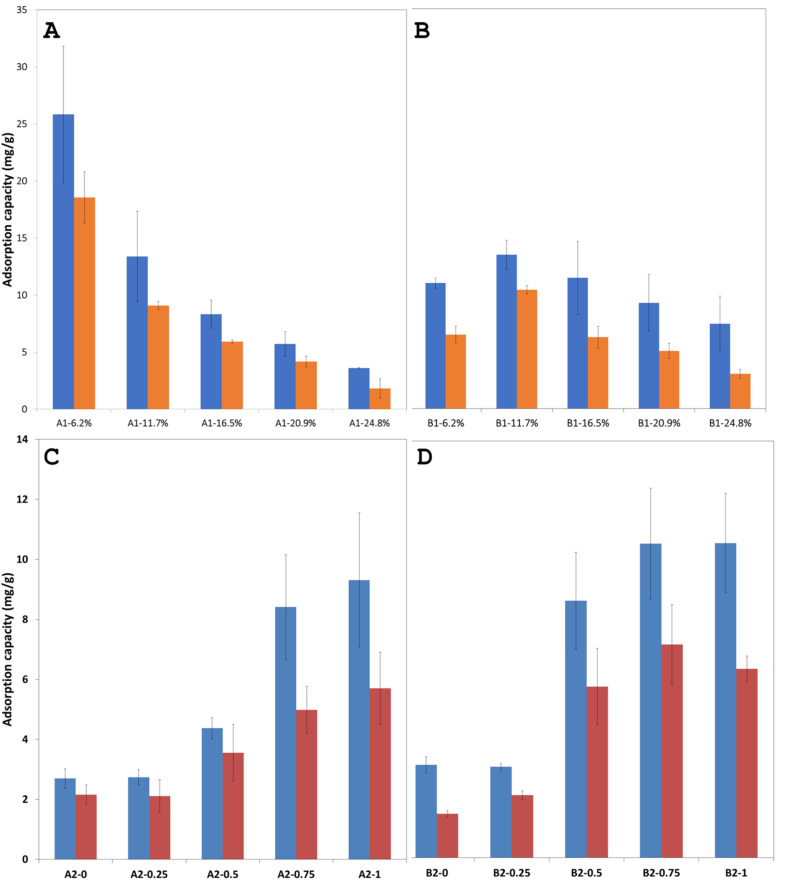
Histogram showing the extraction and recovery capacity of the polymer microspheres for Cu(ii) ions in (A) the homologous series A1 and (B) homologous series B1. Histograms showing the adsorption capacity for Cu(ii) ions for the polymer microspheres in the (C) homologous series A2 and in the (D) homologous series B2.

The experimental results for the Cu(ii) ion extraction capacity *q*_e_ (adsorption of ions into the microspheres) calculated with the eqn (1) and for the recovery capacity *q*_r_ (desorption of ions from the microspheres) calculated with the eqn (2) are given in Fig. 8. Several important trends can be observer. We first note that the adsorption capacities decrease monotonously in the series A1 and series B1, see Fig. 8A and B, emphasizing this way the utmost importance of the cross-linking degree. The drop in the adsorption capacity for the series A1 is much more significant that in the case of series B1, underlining that the nature of the cross-linker plays an important role, with DVB being a stronger crosslinker, *i.e.* closes the pores better than the EGDMA; this statement is supported by comparing the data in the Fig. 4C with the data in Fig. 4D. By closing the pores, the intake of water by the adsorbent decreases drastically. The adsorption and desorption capacity of the Cu(ii) ions for the first few members of the homologous series appear to be significantly larger, almost double, for DVB as a cross-linker, as compared to the case when EGDMA is a cross-linker. However, the last member of both series exhibits a reversed behavior with the capacity of the microspheres when the EGDMA is crosslinker, being almost double than the last members of the series A1 when DVB is the crosslinker, see Fig. 8A and B. Thus, the data imply that the effect of the DVB used in small amounts <20%, is more advantageous for the adsorption capacity than EGDMA, while *vice versa* is true, for large amounts >20%, is disadvantageous when compared to a larger and a more polar molecule crosslinker such as EGDMA.

Here it is important to note that for the series A1 and B1 the evolution of the adsorption *q*_e_ and desorption capacity *q*_r_, Fig. 8A and B correlates directly with the evolution of the pores sizes, Fig. 4C and D, but inversely with the particle size Fig. 4A and B.

Interestingly for the homologous series A2 and homologous series B2 for which the amount of the crosslinker is constant at 16.52% but the amount of the porogen increases from 0 to 1 mL, the evolution is such that the adsorption capacity increases with the increase in the level of the porogen used, see Fig. 8C and D. In other words, an increasing amount of porogen used appears to be beneficial regardless of the crosslinker used. When comparing the adsorption capacity data for both homologous series A2 and B2 they both appear to be very similar, however only very slightly, within the measurement error, more advantageous for EGDMA as a crosslinker at higher toluene amounts.

Here it is important to note that for the series A2 and B2 the evolution of the adsorption *q*_e_ and desorption capacity *q*_r_, Fig. 8C and D, correlates directly with the evolution of the pores size, Fig. 7C and D, and the particle size, Fig. 7A and B.

### 3.3. Competitive adsorption and selectivity in mixed ion matrices Co^2+^ and Cu^2+^

**Fig. 9:**
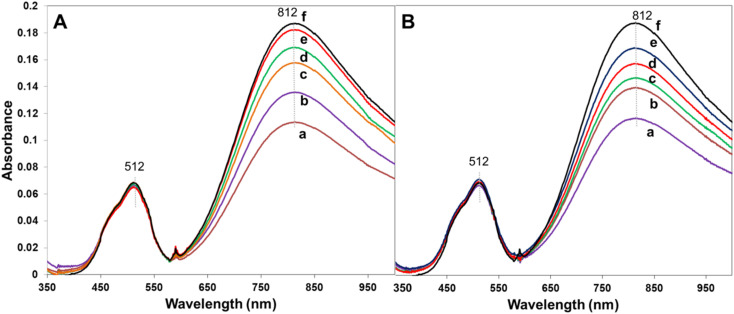
UV-vis adsorption spectra of the solution after being in contact with the absorbent for each of the members of (A) series A1 with DVB as a crosslinker and (B) series B1 with EGDMA as a crosslinker, whereas the curves correspond to the member of the homologous series with (a) 6.2%, (b) 11.7%, (c) 16.5%, (d) 20.9%, (e) 24.8% crosslinker. Curve (f) represents the UV-vis absorption spectrum of the stock solution containing the Cu(ii) and Co(ii) in the same molar concentration.

We have also tested the adsorption and selectivity towards Cu(ii). We have previously reported^11^ that one of the members of the homologous series, *e.g.* A2-0.75, exhibits selectivity toward Cu(ii) when tested in complex metal ion matrices containing Cu(ii), Ni(ii) and Co(ii). Therefore, in the current case we wanted to see if the nature of crosslinker affects the selectivity toward Cu(ii) in more complex matrices, for instance in Cu(ii) + Co(ii), present in the same molar concentration. The UV-vis spectra comparing the intensity of the adsorption band of Cu(ii) at 812 nm and Co(ii) at 512 nm in the stock solution and after being in contact with polymer microsphere adsorbents from the series A1 and series B1 are given in Fig. 9. The data indicate that there is a gradual change in adsorption capacity in the series, namely the adsorption capacity toward Cu(ii) decreases with increase in the level of crosslinker, DVB or EGDMA. However, already qualitatively by visual comparison of the data presented in Fig. 9A*vs.*9B there is no detectable change in the adsorption selectivity with the nature of the crosslinker, judging by the intensity ratio between the absorption bands for Cu(ii) and Co(ii). In other words, none of the microspheres in the series A1 and series B1 adsorb Co(ii), and all of the microspheres in these series adsorb Cu(ii). The observed selectivity for Cu(ii) ions over Co(ii) is attributed to the stronger chemical affinity between Cu(ii) and the nitrogen donor atoms of the pyridine ligand, consistent with known coordination behavior and previously reported results.^11^

### 3.4. Wettability of the homologous series of microspheres

In addition to metal ion adsorption studies we have performed water wettability studies on microparticle powder beds. We have used capillary rise Washburn method^18^ to determine the contact angle of the series of microsphere with the contact angle. We have initially presumed that the contact angle, *i.e.* wettability, may be a correlating factor with the ion-adsorption capacity of the microspheres. Thus, by utilizing Washburn method we have packed samples from both groups of series into a capillary and with the help of a piezoelectric balance of tensiometer we have first acquired the weight of absorbed water in the powder bed *vs.* time curves, from which we have calculated the contact angle utilizing the Washburn equation:^19^3
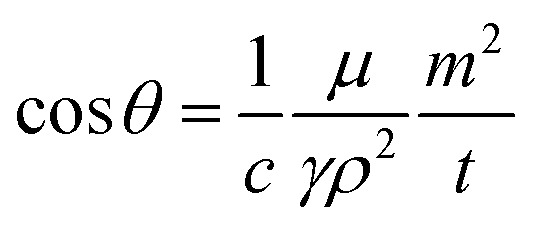
where *m* – is the mass of the fluid measured by a balance, *t* – is time, *γ* – surface tension of the liquid, *ρ* – density of the fluid and *μ* – viscosity and *c* – the capillary constant. Typically, the powder is packed into a glass “capillary” and the intake of water begins upon contacting one end of the capillary tube through a porous frit and monitored with time. The capillary constant, was measured with hexane, for which the above equation was solved for *c* assuming that the *θ* is zero.

**Fig. 10:**
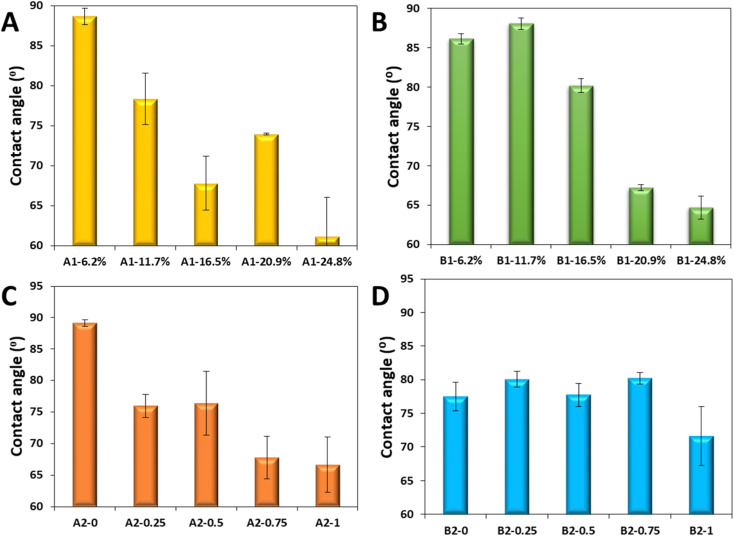
Evolution of the water contact angle with the polymer microspheres from the series A1 (A), series B1 (B), series A2 (C) and series B2 (D).

The results of the water contact angle with the polymer microspheres for all the homologous series are given in Fig. 10.

For the series A1 and B1 the water contact angle decreases with the increase in the amount of crosslinker. Interestingly, the gradual decrease in the contact angle, is directly correlated with the evolution of the pores sizes, Fig. 4C and D, but inversely with the particle size Fig. 4A and B, and directly correlated to the adsorption *q*_e_ and desorption capacity *q*_r_, Fig. 8A and B in the series A1 and B1.

For the series A2 and B2 the water contact angle decreases with the increase in the amount of porogen. This evolution of the contact angle is inversely correlated with the evolution of the pores size, Fig. 7C and D, and also inversely correlated with the particle size, Fig. 7A and B. It is inversely correlated with the evolution adsorption *q*_e_ and desorption capacity *q*_r_, Fig. 8C and D in the series A2 and B2.

From careful examination of these trends we note that the inverse correlation between the water contact angle and the diameter of the polymer microspheres is the only one shared for all series. The rest are switching from one series to the other. This suggests, that the evolution of the water contact angle may be causally determined by the diameters of the microspheres. In other words, the nature of the crosslinker and the porosity influence much less the water contact angle, being a second and a third order contributions, than the diameter of the microparticles. In support of this hypothesis is the discussion of the Kirchberg *et al.*^20^ who found that larger spherical iron–silicon particle sizes yield lower contact angles with the Washburn method; this is exactly as we observe here, the larger the microspheres, Fig. 4 and 7, the lower the contact angles Fig. 10. Although this is a hotly debated topic in literature, whereas some authors declare the Washburn method independent of particle size, at least in the range of 40 to 200 μm, some other authors argue that capillary rise experiments for calculation of the contact angle and wettability behavior of porous medium is a method which depends on the kinetics of flow penetration and the rates of liquid penetration in the powder capillaries as indicated by Washburn equation (eqn (3)). Thus the latter authors concluded from eqn (3) that higher penetration rates increases the value of the slope *m*^2^/*t*, where the other factors are assumed constant, the increase in slope means direct increase in cos *θ* which means a decrease in the contact angle *θ*. In other words if the liquid penetration rate in the powder bed is dependent of other factors as well *m*^2^/*t* = *f*(*a*, *b*, *c*,…), where *a*, *b*, *c*, *etc.* are various parameters, which tend to increase the penetration, consequently the contact angle will appear smaller. The previous conclusion was clearly demonstrated by Kiesvaara & Yliruusi^21^ where they investigated 3 ranges of sieved lactose particle of three size categories >212 μm, 106–212 μm and <106 μm and showed that the liquid penetration rates diminished with diminishing particle sizes; these findings are backed by Galet *et al.*^18^

So, we conclude that particle size is first major parameter that determines the penetration rate, the larger the particles the faster the liquid penetration and thus the lower the contact angle. A second major parameter influencing *m*^2^/*t* (*a*, *b*, *c*,…) is the inner morphology of the particles, or the porosity. By carefully analyzing the data in Fig. 7C and D*vs.* the data in Fig. 10C and D, we note that the evolution of pore sizes, appears to be indeed inversely correlated with the water contact angle, as already noted. However, the evolution of the pore sizes for the series B2 is much more dramatic than for the series A2, the samples from the former series are consistently more porous and with larger pore sizes. On the other hand the decrease in the contact angle with the increase in the sizes of the microspheres for the series B2 is much less obvious than that for series A2; this milder trend cannot be justified alone from the evolution of the diameters of the microspheres in Fig. 7A and B. To explain this softer trend, one must take into account the intake of water by the inner pores of the microspheres, which decrease the penetration rate *m*^2^/*t* of the water into the microparticle bed, increasing the apparent water contact angle as a countertrend set by the decreasing of the contact angle with increasing the diameter of the microspheres. Thus, we conclude that the contact angle determined by Washburn method is also influenced by the increase in the capillarity due to the inner porosity of the microparticles, which has the effect of increasing the contact angle, opposing the decreasing the contact angle due to increase in size of the particles.

A third parameter influencing the contact angle is the surface energy of the material, meaning its surface chemistry and composition, which is the normally the classical factor considered to determine the contact angle of a sessile droplet of liquid onto a perfectly flat surface. In the current case, we note no significant difference in the contact angle with the change in chemistry, of the material although one would expect the EGDMA being more polar than the DVB, the resulting microspheres to also have a lower contact angle, but this is not the case. Thus, we conclude that the major factor contributing to the effective contact angle measured by Washburn is size of the microparticles, followed by the internal pore structure and lastly by the surface energy of the microspheres.

## 4. Conclusions

This investigation has successfully demonstrated the potential of PEmPTech as a versatile and sustainable method for synthesizing polymer microsphere adsorbents and tuning their metal ion adsorption capacity. First, by employing silica nanoparticles for emulsion stabilization in an entirely aqueous environment, we have presented a greener alternative to conventional surfactant-based and organic solvent-based polymerization techniques, typically employed to prepare polymer adsorbents.

In this work, our findings indicate that the adsorption capacity of these microspheres can be effectively tuned through careful and gradual adjustment of crosslinker and porogen levels within the oil phase of the Pickering emulsion droplet. We have thus established clear structure–activity relationships, showing that both the type and amount of crosslinker significantly influence the metal ion adsorption properties of the microspheres. The results from our homologous series highlight that the polymer microspheres' capacity to adsorb Cu(ii) ions improves with increased porogen content, suggesting that porosity plays a critical role in enhancing adsorption performance. Furthermore, the impact of crosslinker type on the physical properties of the microspheres underscores the importance of molecular size and the spatial configuration of the crosslinking agents. Further, although advantageous for chemical stability of polymer, significantly increasing the amount of crosslinker can be detrimental to the adsorption capacity of microparticles. Structural parameters affecting the morphology and size of the particles have no effect on the selectivity for metal ion adsorption underlying the chemical nature of the adsorption selectivity rather than a physical “sieving” of ion through particle morphology. We have also introduced Washburn method as a potential method for gauging “*a priori*” the metal adsorption capacity of the microspheres but no causal relationship has been found. Rather, we clearly established that the water contact angle determined by Wasburn is mainly influenced by the size of the microspheres and secondly by internal porosity. In addition, although the current study focused on the structure–activity relationship, we note that the reusability of similar microspheres synthesized *via* PEmPTech with the same ligand system has been previously demonstrated,^11^ showing good retention of adsorption capacity over fives cycles. Future work will include a detailed investigation of regeneration efficiency and long-term operational stability of the optimized microspheres. Therefore, further studies will focus on application of these homologous series of adsorbents created *via* PEmPTEch for other materials, such as small molecules, dyes, pigments, pollutants to pave the way towards utilization in large scale environmental clean-up and water treatment applications.

## Author contributions

Conceptualization, A. H.; methodology, A. H., O. I. N. and M. H.; validation, A. H., O. I. N. and M. H.; formal analysis, A. H.; investigation, A. H., O. I. N. and M. H.; resources, A. H.; data curation, A. H.; writing—original draft preparation, A. H., M. H.; writing—review and editing, A. H., O. I. N. and M. H.; visualization, A. H.; supervision, A. H.; project administration, A. H.; funding acquisition, A. H. All authors have read and agreed to the published version of the manuscript.

## Conflicts of interest

The authors declare no conflicts of interest.

## Supplementary Material

NA-OLF-D5NA00417A-s001

## Data Availability

Data for this article is available at Open Science Framework at https://doi.org/10.17605/OSF.IO/469G5.
