# Supplementary material for: Optimizing surface properties and particle morphology for metal ion adsorption: precise tuning via Pickering emulsion polymerization
Source: Nanoscale Adv. 2025 Jul 3;7(16):4947–61. doi: 10.1039/d5na00417a (PMC12224202; doi:10.1039/d5na00417a)
Supplement: NA-007-D5NA00417A-s001 [file NA-007-D5NA00417A-s001.pdf]

Supplementary Information (SI) for Nanoscale Advances.
This journal is © The Royal Society of Chemistry 2025

# Supporting Information

## Optimizing Surface Properties and Particle Morphology for Metal Ion Adsorption: Precise Tuning via Pickering Emulsion Polymerization

Andrei Honciuc*, Oana-Iuliana Negru, Mirela Honciuc

*"Petru Poni" Institute of Macromolecular Chemistry, 41A Gr. Ghica Voda Alley, Iasi, 700487, Romania*

* Correspondence: honciuc.andrei@icmpp.ro

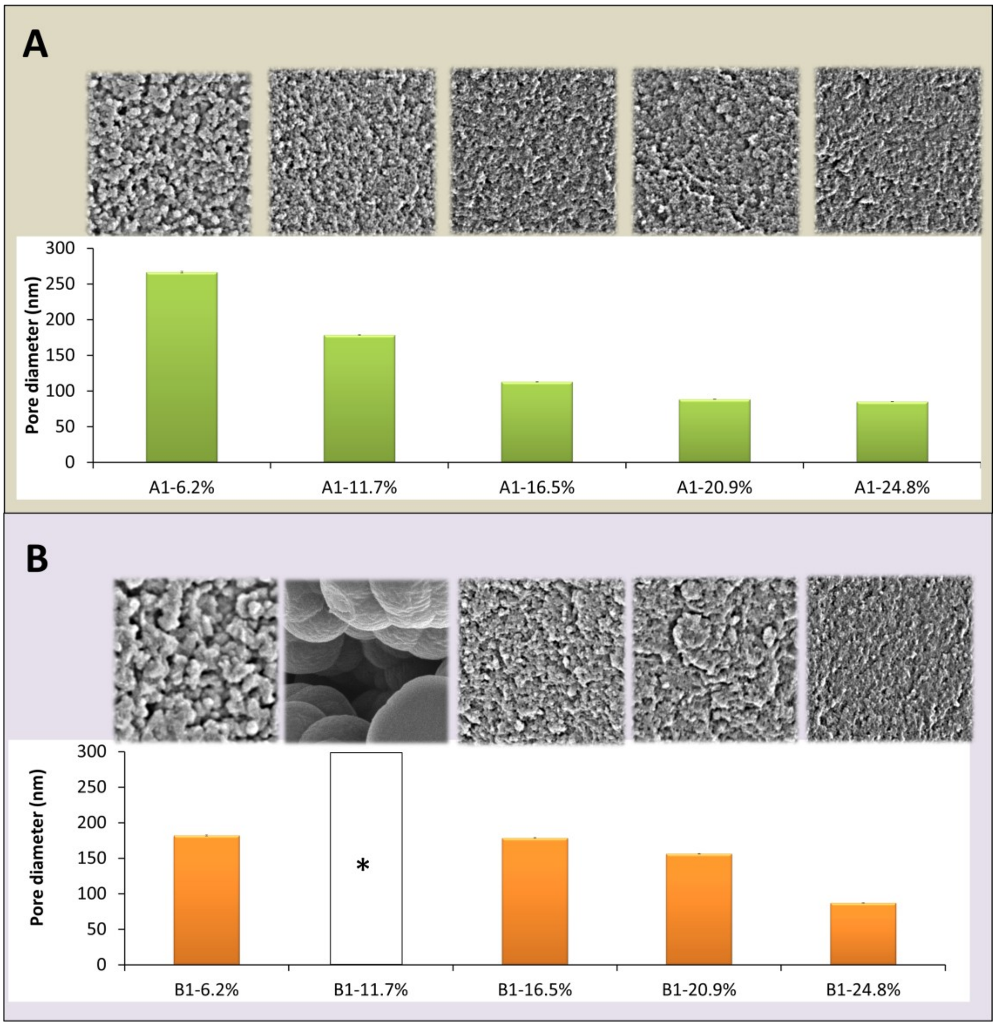


**Figure S1.** SEM images of the cross-section of the Series A1 and Series B1 showing the evolution of the pore size and the corresponding Ferret diameters determined using the ImageJ™ software.

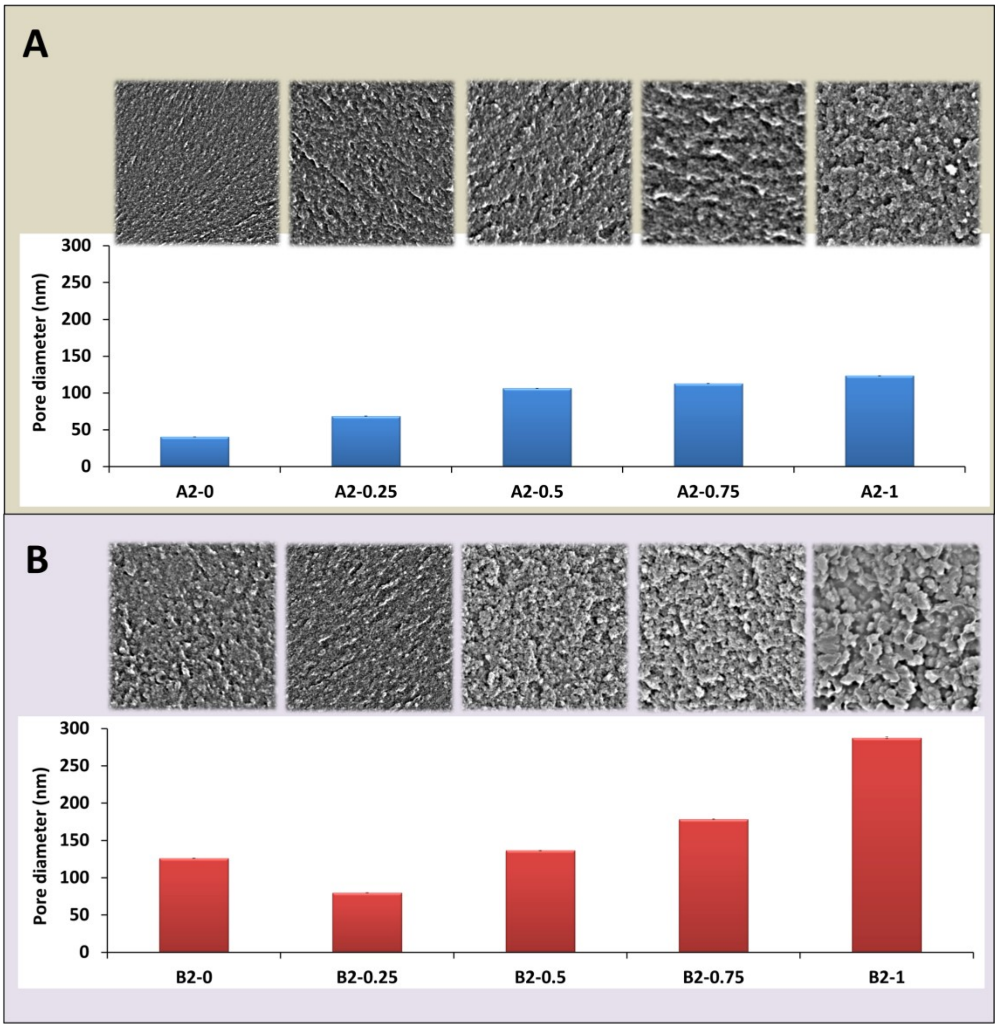


**Figure S2.** SEM images of the cross-section of the Series A2 and Series B2 showing the evolution of the pore size and the corresponding Ferret diameters determined using the ImageJ™ software.
